# Muscle Torque and its Relation to Technique, Tactics, Sports Level and Age Group in Judo Contestants

**DOI:** 10.1515/hukin-2015-0017

**Published:** 2015-04-07

**Authors:** Grzegorz Lech, Wiesław Chwała, Tadeusz Ambroży, Stanisław Sterkowicz

**Affiliations:** 1 Department of Theory of Sport and Kinesiology, University School of Physical Education Al. Jana Pawla II, Cracow, Poland.; 2 Department of Biomechanics, University School of Physical Education, Cracow, Al. Jana Pawla II, Cracow, Poland.; 3 Department of Gymnastics and Dance, University School of Physical Education, Al. Jana Pawla II, Cracow, Poland.

**Keywords:** maximum muscle force, martial arts, course of judo combat, cadets, juniors, seniors

## Abstract

The aim of this study was to perform a comparative analysis of maximal muscle torques at individual stages of development of athletes and to determine the relationship between muscle torques, fighting methods and the level of sports performance.

The activity of 25 judo contestants during judo combats and the effectiveness of actions were evaluated. Maximum muscle torques in flexors/extensors of the body trunk, shoulder, elbow, hip and knee joints were measured. The level of significance was set at p≤0.05; for multiple comparisons the Mann-Whitney U test, p≤0.016, was used. Intergroup differences in relative torques in five muscle groups studied (elbow extensors, shoulder flexors, knee flexors, knee extensors, hip flexors) were not significant. In cadets, relative maximum muscle torques in hip extensors correlated with the activity index (Spearman’s r=0.756). In juniors, maximum relative torques in elbow flexors and knee flexors correlated with the activity index (r=0.73 and r=0.76, respectively). The effectiveness of actions correlated with relative maximum torque in elbow extensors (r=0.67). In seniors, the relative maximum muscle torque in shoulder flexors correlated with the activity index during the second part of the combat (r=0.821).

## Introduction

A number of studies on different sports have demonstrated that an adequate level of muscle strength is required for an effective technical action. Extensive research in this field has been carried out (Jaric et al., 2001; Paasuke et al., 2001; Sleivert et al., 1995). However, the authors primarily emphasized the relationships between the generated muscle torques and sports performance level. It should be stressed that these studies did not adequately consider combat sports and that the individual muscle groups were examined, which does not provide insight into the whole structure of strength abilities of an athlete. The general opinion of judo coaches is that it is technical excellence that primarily contributes to sport results (23.4%) (Sterkowicz et al., 2007). Other factors are psychological and tactical preparation (contributing to 20.1% and 18.0%, respectively). The contribution of physical preparation, which accounts for 29%, includes body constitution and physical fitness factors, with a contribution of 14.8% and 14.2%, respectively.

Assuming that the physical activity of judo contestants is a complex problem, an effective action necessitates an optimal level of physical fitness required for performing particular techniques. A literature survey showed that previous studies on judo technique measured biomechanical characteristics of throws (Blais et al., 2007; Imamura et al., 2006; Imamura and Johnson, 2003). Furthermore, some studies assessed the effectiveness of technique and analyzed the relationships between individual techniques with respect to the direction of throws and the number of scored points (Calmet and Ahmaidi, 2004; Franchini et al., 2008). Temporal structure of the combat was analysed, with regard to time of individual technical and tactical actions (Miarka et al., 2012).

Other studies in elite judo contestants concerned the evaluation of physical fitness in terms of anaerobic and aerobic capacity (Borkowski et al., 2001; Franchini et al., 2011; Little, 1991). Bonitch-Góngora et al. (2012) and Bonitch-Dominguez et al. (2010) examined the effect of fight-induced fatigue on lactate levels, isometric force of upper extremities and peak power of lower extremities, respectively. Successive bouts in judo were demonstrated to diminish maximal isometric strength of both arms (Bonitch-Góngora et al., 2012), but not peak power developed by legs (Bonitch-Dominques et al., 2010).

Apart from the aforementioned studies, the sports performance level has also been measured using morphological variables (Franchini et al., 2005).

The aim of this study was to analyze the level of muscle torques at individual stages of athletes’ development and to determine the relationships between the values of the generated muscle torques and fighting strategies, as well as between muscle torques and the level of performance. The main hypothesis of the study was that the values of maximal muscle force would vary depending on the athlete’s age and should be related to their sports skills level as well as technical and tactical excellence to a different degree.

## Material and Methods

The protocol of the study was approved by the Bioethics Committee of the Regional Medical Chamber in Cracow and complied with the Declaration of Helsinki in terms of the experimental setup and procedures for the use of human subjects in research. Twenty five judo competitors, from cadet, junior and senior age groups, attending six different sport clubs, were enrolled in this cross-section study. The following features were taken into consideration for selection of subjects for testing: age, duration of career, weight category (the lightest and the heaviest category were not included) and sports skills level (each of the subjects ranked at least fifth in the national competitions). Table 1 provides the demographical, sports career and key body constitution characteristics of the participants.

Tests in the individual age groups were planned in order to register the competitive activity of the contestants during the main competition of the season, and to determine muscular torques immediately after the competition.

A video recording of the combats was made in the individual age groups:

- seniors: 3rd and 4th round of the Individual-Team Seniors League,
- juniors – qualifying combats in the Junior Poland Cup, and the Junior Poland Cup,
- cadets – qualifications to the Poland Youth Olympics and the Poland Youth Olympics.

The study analyzed only the qualifying combats between the contestants who qualified for national competitions. In total, 175 bouts were recorded for the analysis (51 senior, 58 junior and 66 cadet combats). A computer-aided analysis was employed for the recorded technical actions of individual competitors. Actions that scored points and the part of the bout where technical action was performed were taken into consideration. The analysis also included ineffective actions, which did not result in scoring points. This occurred when a competitor threw his opponent off balance when attempting a throw (the so-called flight phase was observed). Such actions were given a 0 mark. An overall of 373 technical actions were registered. The observations and analysis of the registered judo bouts were carried out by two experienced coaches. The objectivity of the observations was very high. Inter-observer coefficients between both activity index (WA) and effectiveness index (WS) were 0.993, 95% CI: 0.968–0.998 and 0.997, 95% CI: 0.988–0.998, respectively. Similarly, the reliability determined by intra-class correlation coefficients (ICC) of WA and WS indices was very high (SEM was 3.3% and 2.4%, respectively). For two measurements of the same evaluator, ICC values were 0.991, 95% CI: 0.970–0.998 for WA and 0.996, 95% CI: 0.984–0,999 for WS. The ICC ratio measures the relative homogeneity within groups in relation to the total variation. The bout was divided into two parts. In the senior group, the first part was represented by the first three minutes, whereas the second part was represented by the fourth and fifth minutes (the effective time of the bout being 5 minutes). For juniors and cadets, the first part of the combat was represented by the first two minutes whereas the second part was represented by the third and fourth minute (the effective time of the combat being 4 minutes). If extra time was applied, it was included in the second part of the bout. Based on the collected data, indices that determine the activity and effectiveness of actions among the study participants were calculated. The activity index (WA) was calculated using the following formula:

(1) WA=ΣA/NW,

where ΣA is a total of the attacks and NW is the number of judo combats the contestant took part in. The WA1 and WA2 were the activity indices calculated for the first and second parts of the bout, respectively. Another index, RWA (difference in the activity index), was also calculated to reflect the variability of activity during competition. It was calculated as follows:

(2) RWA=WA1−WA2,

The effectiveness index (WS) is an arithmetic mean of the notes for attacks (WS1 as calculated for the first part of the bout and WS2 for the second part). The difference in the effectiveness index was calculated from the following formula:

(3) RWS=WS1−WS2,

The sports level was evaluated according to the following point scale:

- qualifying tournaments: 1st place – 3 points, 2nd place – 2 points, 3rd place – 1 point, 5th place – 0.5 points; and
- national competitions: 1st place – 7 points, 2nd – 5 points, 3rd – 3.5 points, 5th – 1.5 points, and 7th – 0.5 points.
- The evaluations of muscle torques were conducted in 2006. They included measurements of maximum muscle force in flexors and extensors of the trunk, shoulder, elbow, hip and knee joints. The maximal muscle force generated during isometric contraction was evaluated using measurement stations in standard positions (Table 2). The levels of maximum and relative torques were calculated based on the obtained force values from the following formula:
  (4) τmax=Fmax d,
  where τmax is the maximum torque in the tested muscle group [Nm], Fmax is the maximum force developed during isometric contraction in the tested muscle group [N], and d is the moment arm for the external force (the distance from the biomechanical rotation axis in the joint to the line of the dynamometer) [m].
  The relative values of torques were calculated from the formula:
  (5) τr=τmax/m,
  where: τr is the relative torque [Nm/kg] and m is body mass of a competitor [kg].

Force measurements were carried out in a measuring setup composed of a Hottinger tensometric sensor (accuracy: 0.5%), an AD card and a PC. The data were stored and analyzed in the AAD Analog Signal Recorder application (developed by the Nuclear Physics Department of the Polish Academy of Science, Cracow, Poland). Before the maximum isometric contraction was performed, a subject was stabilized on the measurement platform with locking mechanisms in order to ensure accurate measurements in these positions.

The STATISTICA.PL software was used for data analysis. The Shapiro-Wilk test was used to study the normality of distribution. The homogeneity of variance was tested with the Levene’s test. Kruskal-Wallis tests H and F-value were used in the analysis of variance, according to the distribution of the sample and homogeneity of variance. The Tukey-Kramer and Mann-Whitney tests were used to study the differences between the mean values of individual groups. The Mann-Whitney test with the Bonferroni correction was also employed. It divided the significance level p=0.05 by the number of comparisons. The correlation analysis was based on the Spearman’s rank correlation coefficient (RSp).

## Results

Table 3 shows the values of indices characterizing the course of the judo bouts and sports performance levels.

The Tukey-Kramer procedure carried out for the relative torque in the elbow flexors found the groups of seniors and juniors were homogeneous (Table 4). It was observed that the highest levels of this index were in the senior and junior groups, whereas lower values were found in the cadet group. Comparison of mean values obtained for shoulder extensors and trunk extensors in the senior and cadet groups indicated that those groups were homogeneous in regard to these variables. Significantly higher values of these indices were obtained in the senior group, while lower values were found in the junior group and the cadet group. With regard to mean values of hip extensors (HJ EXT) and total torque, two pairs of homogeneous groups were created based on the comparison of mean values: (1) seniors and cadets and (2) juniors and cadets. The highest mean values of these indices were found in the senior group. They were significantly higher than the lowest, mean values recorded in the junior group. Comparison of the means in trunk flexors revealed homogeneity of the senior and cadet groups. Higher means were found in the senior and cadet groups. The lowest values were calculated in the junior group.

Table 5 includes statistically significant levels of the rank correlation coefficient calculated between the torques of the tested muscle groups, and the indices of fighting methods together with the level of performance in the senior group. All cases were highly correlated (0.7 ≤ RS p<0.9).

A positive relationship was found between:

- The competitors’ activity (WA) and the muscle torque in hip extensors (HJ EXT);
- The differences between the activity index (RWA) and muscle torque in trunk extensors (TR EXT).

A negative correlation was found between:

- The effectiveness of actions in the first part of the bout and muscle torque in the hip extensors;
- The effectiveness of actions in the second part of the bout and muscle torque in hip flexors;
- The level of performance (PO) and muscle torque of shoulder extensors.

In the junior group (Table 5), a very high positive correlation between WA and EJ FLX was observed (muscular torques of the elbow flexors). A high negative correlation was found between WA1 and SJ EXT. The activity during the second part of the bout (WA2) significantly correlated with strength abilities of three muscle groups. It correlated positively with KJ FLX, but negatively with HJ FLX and HJ EXT. A strong negative correlation was observed between RWA and KJ FLX. The effectiveness of actions was associated with two muscular torque values: a high positive correlation was found for its relationship with EJ EXT, while a high negative one was observed for TR EXT. The effectiveness of actions in the first part of the bout correlated with SJ FLX (a high negative correlation).

In the cadet group (Table 5), a high correlation between the activity of the contestants in the second part of the judo bout and muscle torque in shoulder extensors was found. The effectiveness of actions correlated (a strong negative correlation) with TR EXT. The effectiveness in the first part of the bout was associated with the three maximum muscular strength indices: SJ FLX, TR EXT and total torque. A high negative correlation was found in all cases (0.7 ≤ RSp <0.9).

## Discussion

Analysis of literature which has focused on the effect of muscle force on activity of judo contestants reveals that few publications contain significant information which combines these two topics. Although Blais et al. (2006; 2007) determined the level of relative force generated by contestants when performing throws with a partner or a judo specific device equipped with force sensor, the level of generated force was not compared to sports skills level. However, interesting findings of these authors concern significantly lower values of relative strength recorded during exercises with a partner compared to the same throws performed using a judo specific device. In another study, Iwai et al. (2008) compared the level of back extensor and flexor muscle strength with the sports level in judoists and wrestlers. The study lacked evaluation of a broader range of muscle groups which might have an effect on judoists’ activity. It should be emphasized, however, that Iwai et al. (2008) did not find significant relationships between back muscle torques and the sports performance level of the subjects. Furthermore, they stressed that both sports required a specific method of development of muscle strength. In judo, it should be oriented towards using back muscle strength mainly to perform rotation movements and swaying to the sides.

When comparing relative muscle torque in five of the investigated muscle groups (EJ EXT, SJ FLX, KJ FLX, KJ EXT, HJ FLX) between all the analyzed groups of contestants, no significant differences were found (p<0.05). Non-significant differences between seniors and cadets were also found in three other indexes: HJ EXT, TR FLX and maximal muscle torques. This fact points to the phenomenon observed in training practice, i.e. early specialization, which consists of the application of the senior training model in younger age groups, with the focus on high sport results within a short time. Hence, both natural mechanisms and biological principles of physical development, as well as the care for future sports performance are ignored.

Significant correlations between the indices of technical and tactical excellence of the contestants found in the present study and the level of measured maximal muscle torques provide a relatively complex model of interactions. High correlations (RSp=0.82) between hip joint extensors and the activity during a bout in the group of seniors were found in the present study. These antigravity muscles are more intensively used during most of throws in competitive judo (Imamura and Johnson, 2003). However, levels of hip extensor and flexor muscle forces negatively correlated with the effectiveness in the first and second part of the judo bout, respectively. This also suggests that the contestants who have strong hip extensors and flexors focus their actions on high activity, which is frequently not accompanied by high effectiveness. Reduction in the activity of contestants in the second part of the bout, reflected by the RWA index, shows a strong positive relationship with the level of trunk extensor torques. This might suggest that the contestants who use trunk extensors extensively and frequently during the first part of the match fail to maintain a high level of activity during the second part of the combat due to increasing fatigue in these muscle groups. A negative significant correlation between torques of SJ EXT and the level of performance should also be discussed. The contestants who had excessively developed SJ EXT extensors usually showed a lower level of performance. In juniors, values of elbow and knee flexors’ torques correlated positively with activity, whereas elbow extensors correlated with effectiveness. With regard to knee extensors, taking into consideration the most frequently used techniques (hand throwing techniques), the phenomenon of preparation for the main attack is likely to occur. Similar interpretation can be employed for the correlation between the values of elbow extensor torques and the effectiveness index. Elbow flexors are muscles which also determine the effective phase of Kuzushi – i.e. the first and basic phase of each throw, which determines its quality. Comparison of the results obtained in the group of seniors with the group of juniors reveals that the topography of muscle groups which affect both the activity and the effectiveness of a judo bout is being remodelled. Observation of the development of strength abilities in contestants across different age categories reveals higher and more favourable effects of large muscle groups, e.g. HJ on the course of a judo bout. Cadets demonstrated a positive correlation with elbow extensors and activity during the second part of the bout. Mean values of maximal muscle torque in SJ EXT in the group of cadets correlated positively with the activity during the second part of the combat. These muscles, similarly to elbow flexors, are a precondition for the effective performance of the Kuzushi phase of throws. Other relationships in the analyzed muscle torques and assessment of the effectiveness point to a negative effect of the level of strength abilities on the quality of performing throws.

In summary of the assessment of the effect of individual muscle groups on the effectiveness and activity during a judo bout, a substantial number of negative relationships was found for different age categories. This indirectly suggests the need for selective development of muscle strength in those muscle groups which determine high values of the analyzed indices. However, this does not mean that the development of other muscles should be neglected, but it must be carried out in a harmonious manner, aimed at optimization of the strength level so that it does not inhibit the development of technique, with particular focus on younger age groups where “early specialization” should be avoided. Similar suggestions were expressed by Iwai et al. (2008).

Assuming that the likelihood of being successful in fighting with individual opponents is determined by a comparable level of physical preparation (Calmet and Ahmaidi, 2004) and taking into consideration the results of the present study, one can propose practical implications for strength training in judoists. It is difficult to enhance the development of technical and tactical excellence and, consequently, fighting tactics, merely on the level of maximal strength in each age group. Notably, only few correlations turned out to be statistically significant, while most of them were negative. The relationships between the sum of maximal muscle torques and the level of sports performance were not found to be significant in any of the analysed age groups. With respect to the analysis of the results of our study and reports by other authors (Borkowski et al., 2001; Franchini et al., 2005; 2011), it seems that other indices of preparation in judo contestants, connected with the course of the bout and their sports level, should be considered and included in coaching practice.

## Conclusions

1. No differences in maximal muscular torques were found between the studied groups of contestants for elbow extensors, shoulder flexors, knee flexors and extensors and hip flexors. The mean values obtained for hip extensors and the total torque show the highest levels of these indices in the senior group. They significantly differed from the levels observed in the juniors, in whom the lowest values were obtained.
2. The highest mean values of torques in shoulder and trunk extensors were found in the senior group. Lower means were found in the groups of juniors and cadets, who formed a homogeneous group. In case of elbow flexors, higher mean values were calculated in senior and junior groups, whereas lower mean values were found in cadets. Mean values obtained for trunk flexors were significantly higher in seniors and cadets who formed a homogeneous group.
3. Maximal muscle torque values did not correlate significantly with sports performance levels in elite judo contestants.
4. Specific, individual physical characteristics of judo contestants should be considered in the process of developing their technical and tactical performance.

## Figures and Tables

**Table 1:** Basic characteristics of the study participants

	Senior (1)		Junior (2)		Cadet (3)	
	N	mean (±SD)	N	mean (±SD)	N	mean (±SD)
Age (years)	7	21.6±0.98	10	17.5±0.71	8	15.5±0.54
training experience (years)	7	12.4±1.7	10	8.4±1.2	8	6.1±0.8
Body height (cm)	7	180.4±5.4	10	180.4±3.6	8	177.4±6.2
Body mass (kg)	7	82.2±7.6	10	85.8±10.5	8	71.7±7.5
Lean body mass (kg)	7	74.2±6.2	10	72.1±5.5	8	65.2±6.3

**Table 2:** Standard joint positions for measurements of maximal muscle torques

Measured muscle group	Measurement position	Angles in the joints between adjacent segments		
τmax FLX and EXT SJ	standing	SJ - 0 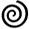	EJ - 0 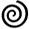	WJ - 0 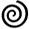
τmax FLX and EXT EJ	standing	SJ - 0 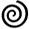	EJ - 90 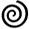 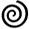 (flex)	WJ - 0 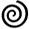
τmax FLX and EXT HJ	supine	HJ - 90 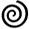 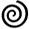 (flex)	KJ - 90 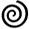 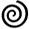 (flex)	AJ - 0 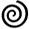
τmax FLX and EXT KJ	Sitting	HJ - 90 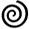 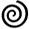 (flex)	KJ - 90 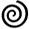 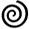 (flex)	AJ - 0 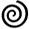
τmax FLX and EXT TR	Sitting	HJ - 90 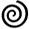 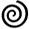 (flex)	KJ - 90 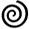 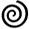 (flex)	AJ - 0 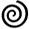

FLX – flexors, EXT – extensors, SJ – shoulder joint, EJ – elbow joint, WJ – radiocarpal (wrist) joint, HJ – hip joint, KJ – knee joint, AJ – ankle joint TR – trunk, Angle 0º, 90º– Angles between neighboring body segments in measurement positions. According to judo match evaluators mentioned above, the muscles which were mostly engaged in the Seoi-nage throw were SJ FLX, TR EXT, EJ FLX, HJ EXT, KJ EXT, and TR FLX.

**Table 3:** Technical and tactical excellence indices and the level of performance in the studied groups of judo competitors

	
	Senior mean (±SD)	Junior mean (±SD)	Cadet mean (±SD)
WA	2.63±0.72	2.13±0.63	1.73±0.76
WA1	1.8± 0.60	1.03±0.45	1.16±0.61
WA2	1.37±0.28	1.56±0.56	0.94±0.59
RWA	0.43± 0.67	− 0.53±0.73	0.57±0.42
WS	2.84±1.00	3.42±1.37	4.51±1.05
WS1	3.10±1.77	4.04±2.02	5.25±1.76
WS2	2.11±1.24	3.24±2.06	2.96±2.71
RWS	1.00±2.14	0.81±2.95	2.30±4.20
PO	4.00±5.07	3.30±1.51	2.88±3.01

**Table 4:** Mean values of relative muscle torques (Nm/kg) in different age groups of contestants

	Senior (1) mean (± SD)	Junior (2) mean (± SD)	Cadet (3) mean (± SD)
EJ FLX^**^ (elbow flex.)	1.19 ± 0.19 (3^*^)	1.29 ± 0.15 (3^**^)	0.93 ± 0.13
EJ EXT (elbow ext.)	0.74 ± 0.13	0.87 ± 0.12	0.88 ± 0.25
SJ FLX (shoulder flex.)	1.68 ± 0.32	1.62 ± 0.26	1.58 ± 0.29
SJ EXT^**^ (shoulder ext.)	1.75 ± 0.30 (2^##^, 3^##^)	1.09 ± 0.13	1.21 ± 0.11
KJ FLX (knee flex.)	1.31 ± 0.29	1.58 ± 0.23	1.54 ± 0.25
KJ EXT (knee ext.)	3.41 ± 0.67	3.75 ± 0.51	3.44 ± 0.59
HJ FLX (hip flex.)	2.02 ± 0.16	1.64 ± 0.45	1.77 ± 0.58
HJ EXT^*^ (hip ext.)	4.78 ± 0.31 (2^##^)	3.31 ± 0.85	4.02 ± 0.97
TR FLX^**^ (trunk flex.)	3.02 ± 0.09 (2^##^)	1.48 ± 0.21 (3^##^)	2.72 ± 0.70
TR EXT^**^ (trunk ext.)	6.03 ± 0.30 (2^##^, 3^#^)	3.84 ± 0.29	4.74 ± 1.21
Torque total ^**^	25.93 ± 1.30 (2^##^)	20.47 ± 1.15	22.84 ± 2.83

Indices referring to the measurements of the elbow, shoulder, hip and knee joints: EJ FLX, EJ EXT, SJ FLX, SJ EXT, KJ FLX, KJ EXT, HJ FLX, HJ EXT are the arithmetic means of the right and left joints.

*: p<0.05;

**: p<0.01.

For multiple comparisons used with the Mann-Whitney test -

#: p<0.016;

## p<0.01.

**Table 5:** Statistically significant values of the correlation coefficient between torques in the tested muscle groups, and the indices of fighting methods and the level of performance in the seniors, juniors and cadets

	N	R Spearman	t(N-2)	p
Senior				
**WA & HJ EXT**	**7**	**0.821**	**3.221**	**0.023**
RWA & TR EXT	7	0.883	4.205	0.008
WA1 & HJ EXT	7	−0.786	−2.840	0.036
WA2 & HJ FLX	7	−0.786	−2.840	0.036
PO & SJ EXT	7	−0.829	−3.313	0.021
Junior				
**WA & EJ FLX**	**10**	**0.730**	**3.022**	**0.017**
WA1 & SJ EXT	10	−0.784	−3.574	0.007
**WA2 & KJ FLX**	**10**	**0.757**	**3.277**	**0.011**
WA2 & HJ FLX	10	−0.739	−3.098	0.015
WA2 & HJ EXT	10	−0.751	−3.216	0.012
**RWA & KJ FLX**	**10**	**−0.745**	**−3.163**	**0.013**
**WS & EJ EXT**	**10**	**0.673**	**2.572**	**0.033**
WS & TR EXT	10	−0.758	−3.283	0.011
WS1 & SJ FLX	10	−0.657	−2.462	0.039
Cadet				
**WA2 & SJ EXT**	**8**	**0.756**	**2.832**	**0.030**
WS & TR EXT	8	−0.810	−3.378	0.015
WS1 & SJ FLX	8	−0.778	−3.038	0.023
WS1 & TR EXT	8	−0.755	−2.816	0.031
WS1& TORQUES TOTAL	8	−0.743	−2.715	0.035

Favorable correlations are written in bold (higher level of muscular torque correlated with favorable levels of the indices of fighting methods, or the contestants’ level of performance).
